# Perceptual Properties of the Poisson Effect

**DOI:** 10.3389/fpsyg.2020.612368

**Published:** 2021-01-22

**Authors:** Takahiro Kawabe

**Affiliations:** Human Information Science Laboratories, NTT Communication Science Laboratories, Tokyo, Japan

**Keywords:** material perception, the Poisson effect, force direction judgment, image deformation, elastic materials

## Abstract

When an elastic material (e.g., fabric) is horizontally stretched (or compressed), the material is compressed (or extended) vertically – so-called the Poisson effect. In the different case of the Poisson effect, when an elastic material (e.g., rubber) is vertically squashed, the material is horizontally extended. In both cases, the visual system receives image deformations involving horizontal expansion and vertical compression. How does the brain disentangle the two cases and accurately distinguish stretching from squashing events? Manipulating the relative magnitude of the deformation of a square between horizontal and vertical dimensions in the two-dimensional stimuli, we asked observers to judge the force direction in the stimuli. Specifically, the participants reported whether the square was stretched or squashed. In general, the participant’s judgment was dependent on the relative deformation magnitude. We also checked the anisotropic effect of deformation direction [i.e., horizontal vs. vertical stretching (or squashing)] and found that the participant’s judgment was strongly biased toward horizontal stretching. We also observed that the asymmetric deformation pattern, which indicated the specific context of force direction, was also a strong cue to the force direction judgment. We suggest that the brain judges the force direction in the Poisson effect on the basis of assumptions about the relationship between image deformation and force direction, in addition to the relative image deformation magnitudes between horizontal and vertical dimensions.

## Introduction

Human observers can easily distinguish rigid and non-rigid materials. Previous vision science literature has shown that spatial motion patterns are diagnostic features for an observer to perceive non-rigid materials. For example, Jain and Zaidi (2011) argued that the brain calculate an object non-rigidity from motion shears. Kawabe et al. (2015) showed that the smoothness of the spatial motion pattern was an important feature for liquid perception. Bi et al. reported that spatial motion patterns determine the perception of fabric stiffness (Bi and Xiao, 2016; Bi et al., 2018, 2019). Several previous studies (Paulun et al., 2017; Schmidt et al., 2017; Schmid and Doerschner, 2018) have shown that optical, shape, and motion cues were selectively and/or interactively used to estimate object stiffness. Although these studies emphasize the importance of imaging features, it is still unclear what kind of material mechanical behavior is associated with which imaging features in spatial motion patterns.

**FIGURE 1:**
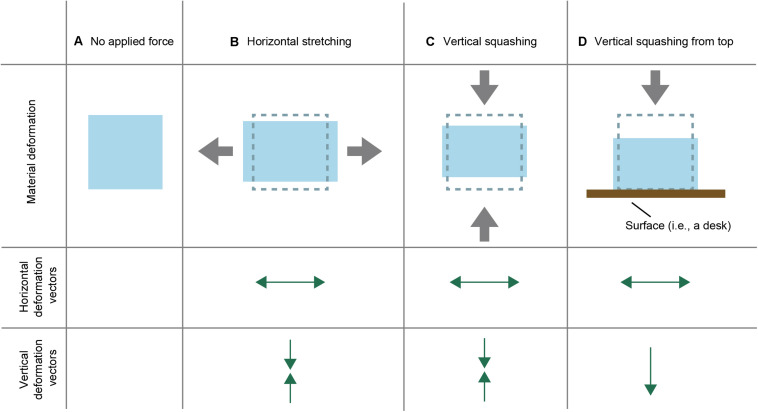
Schematic explanation of the two-dimensional Poisson effect. **(A)** A two-dimensional depiction of an elastic material. **(B–D)** Schematic depictions about how the material **(A)** deforms under **(B)** horizontal stretching, **(C)** vertical squashing, and **(D)** vertical stretching from the top. Each depiction is accompanied by related horizontal and vertical deformation vectors.

This study focuses on the simple but important physical behavior of materials, the so-called Poisson effect. When an external force is applied to an elastic material (Figure 1A), the material behaviors uniquely in its mechanical properties. For example, when a fabric material is stretched (or squashed) horizontally, it is vertically compressed (or extended) at the same time. Figure 1B schematically illustrates a typical situation of a Poisson effect in which the material is stretched horizontally while compressed vertically. This physical phenomenon, which is observed in natural elastic materials, is called the Poisson effect. The Poisson effect can be quantitatively described with the Poisson’s ratio ν, which is defined by the following formula,

(1) $$\nu =-\left(d_{\text{y}}/\text{h}\right)/\left(d_{\text{x}}/\text{w}\right),$$

wherein w is the length of the material along the longitudinal axis of strain, h is the length of the material along the transverse axis of strain, *d*_*x*_ is the change in length due to longitudinal strain and *d*_*y*_ is the change in length due to transverse strain (Poisson, 1827; Greaves et al., 2011). The Poisson’s ratio is different from material to material. For example, the Poisson’s ratio of natural rubber is nearly 0.49, while the Poisson’s ratio of cork is nearly 0. It is known that the Poisson’s ratio of natural materials does not exceed 0.5.

A matter of concern in perception science is whether and how the human observers predict and discriminate physical events (Kaiser et al., 1992; Battaglia et al., 2013; Ullman et al., 2017). To examine the visual cue to the Poisson effect, Kawabe (2019) asked observers to judge whether the horizontal stretching of material was causally related to its vertical compression. The researcher observed that the causality was often reported even when the simulated Poisson’s ratio exceeded 0.5, suggesting that the observers do not rely their judgments on the physical fidelity of the Poisson’s ratio. Rather, Kawabe (2019) discussed that human observers judged the causality on the basis of image features related to material deformation. Under the different scenario, Kawabe (2020) found that the Poisson’s ratio modulated the softness perception in the pseudo-haptic feedback. In the researcher’s experimental setting, a participant moved their hands in front of an LCD monitor displaying an elastic material. The material deformed in accordance with the position of participants’ hands while an external device tracked the positions of the hands. When the participant conducted horizontal hand movements as if they horizontally stretched a material in the air, the material in the display was horizontally stretched. At the same time, the material was also vertically compressed on the basis of assumed Poisson’s ratio. The participants reported the subjective stiffness of the material in a five-point scale. Kawabe (2020) found that the material with the higher Poisson’s ratio was reported to be softer than the material with the lower Poisson’s ratio. The results are consistent with previous studies showing that the stiffness of an elastic material is judged on the basis of the image statistics of image motion/deformation (Kawabe and Nishida, 2016; Bi et al., 2019).

The present study conducted an online experiment to examine how the observers distinguished stretching from squashing in the Poisson effect. In three-dimensional materials, the Poisson effect is related to three spatial axes (that is, a longitudinal axis and two transverse axes). On the other hand, if the material is thin, the secondary horizontal axis can be ignored. From this point of view, two-dimensional stimuli including deformation along two spatial axes were used as stimuli in this study. As described above, the horizontal stretching of an elastic material causes the vertical compression in one situation of the Poisson effect. On the other hand, in the other situation of the effect, the vertical squashing of an elastic material causes a horizontal expansion. Importantly, in both situations, the Poisson effect involves an image deformation with horizontally outward and vertically inward vectors (please compare Figure 1B with Figure 1C). The relative deformation magnitudes between horizontal and vertical dimensions should be a clue to the discrimination between stretching and squashing. In the Poisson effect with natural materials, when an elastic material is horizontally stretched, the magnitude of the horizontal stretching is greater than the magnitude of the vertical compression (because as described above the Poisson’s ratio for natural materials does not exceed 0.5). On the other hand, when an elastic material is vertically squashed, the magnitude of the horizontal extension is smaller than the magnitude of the vertical compression. Thus, we wanted to confirm that the relative magnitude of deformations between the vertical and horizontal dimensions would determine the observers’ judgment as to whether the material was stretched or squashed. Additionally, we explored the effect of the direction of stretching/squashing on the judgment. In the previous studies (Kawabe, 2019, 2020), the perception of the Poisson effect was examined by using the stimuli wherein a material was horizontally stretched. In this study, we examined whether there are differences in judgment between the horizontal and vertical stretching/squashing situations of a material.

The main purpose of the present study was to check whether the asymmetric pattern of deformation influenced the stretching-squashing judgment. In the typical explanation of the Poisson effect, it is assumed that an external force is symmetrically applied to both the top and bottom (or left and right) surfaces of an elastic material. However, in the real-world situation, the material to which an external force is vertically applied is typically located on a plane (e.g., a floor). Under the scenario, the material deformation is expected to be anisotropic (Figure 1D). Specifically, when an external force is vertically applied to the top of the material, the bottom of the material does not deform upward. If the brain took advantage of the anisotropic deformation as a cue to the judgment of the Poisson effect, the anisotropic deformation, as shown in Figure 1D, would bias stretching-squashing judgment in the direction of squashing.

## Materials and Methods

### Observers

In total, 822 people participated in the experiment. Their mean age was 40.1 (SD11.33). They were recruited online by a crowdsourcing research agent in Japan and paid for their participation. Only the people who could participate in the experiment using their personal computers were recruited. They were unaware of the specific purpose of the experiment. Ethical approval for this study was obtained from the ethics committee at Nippon Telegraph and Telephone Corporation (Approval number: R02-009 by NTT Communication Science Laboratories Ethics Committee). The experiments were conducted according to principles that have their origin in the Helsinki Declaration. Written informed consent was obtained from all observers in this study.

### Stimuli

**FIGURE 2:**
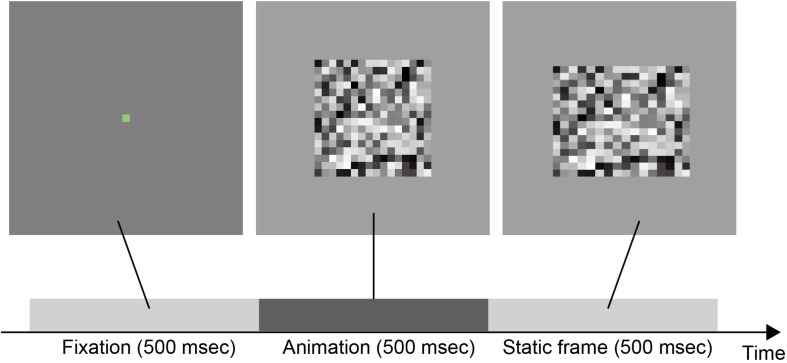
A schematic diagram of stimulus sequence on a single trial.

Stimuli were movies, each of which consisted of a fixation followed by the animation of deforming a two-dimensional grayscale random noise square (see Figure 2 and Supplementary Video 1). The stimuli subtended 512 × 512 pixels. The fixation was a green (RGB value: R:0, G:255, B:0) square subtending 16 × 16 pixels and presented for 500 msec (i.e., 30 frames). After the fixation, the animation of the deforming square was presented for 500 msec (i.e., 30 frames) and followed by the static final frame of the animation.

#### Noise Square

The noise square subtended 256 × 256 pixels. The size of each noise cell was 16 × 16 pixels, and hence, the noise square consisted of 16 × 16 noise cells. The intensity of each noise cell was randomly chosen from the uniform distribution ranging between 0 and 255.

#### Deformation

**FIGURE 3:**
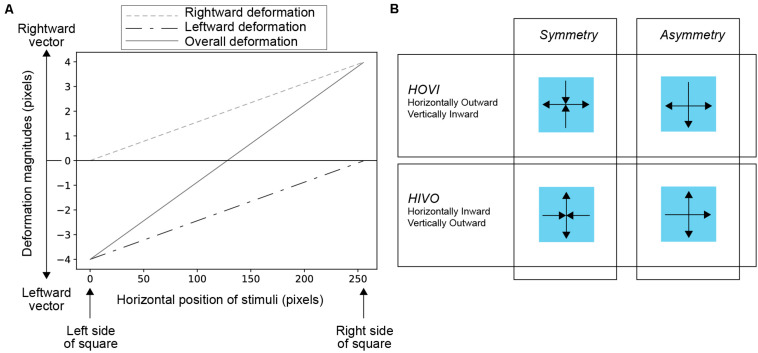
**(A)** The manipulation of deformation magnitudes across space. The graph shows the condition with the *d*_*max*_ of four pixels. The horizontal axis indicates the horizontal position of the square in the stimulus. The values 0 and 255 indicate the left and right side of the square, respectively. The vertical axis indicates the magnitude of the deformation in pixels. Positive and negative values indicate the magnitude of the vector in the right and left directions, respectively. In the image deformation of the stimulus, pixels were displaced by the vectors. Dashed and dotted-dashed lines indicate spatial variations in the amount of deformations in the right and left directions, respectively. When you merge the two lines, you get a solid line of overall deformation; **(B)** Schematic explanations of Symmetry and Asymmetry deformation vectors in HOVI (Horizontally-Outward-Vertically-Inward) and HIVO (Horizontally-Inward-Vertically-Outward) conditions.

The noise square deformed in the horizontal as well as vertical directions. To deform the square, we used a conventional pixel warp method with a linear interpolation of pixel values (Glasbey and Mardia, 1998). Figure 3A shows how the deformation magnitude (that is, the magnitude of pixel warp) varies across the horizontal dimension. The rightward and leftward deformations were controlled by spatially linear functions (a dashed line and a dash-dotted line in Figure 3A, respectively), each of which involved with the opposite vector of deformation. As shown as a solid green in Figure 3A, the overall deformation patterns were drawn from the summation of the two functions. The manipulation of the spatial properties of deformation was based on Kawabe (2019). In generating the animation of the deforming square, the maximum amplitude of deformation (*d*_*max*_) was first determined. Second, the deformation magnitude of the square on the *n*th frame of the animation (*d*_*n*_) was determined by the following formula,

(2) $$d_{n}=d_{\text{max}}\times \text{sin}\,\left(2\,\pi \,n/120\right),$$

wherein *n* ranged from 1 to 30. As shown in Table 1, the *d*_*max*_ was determined separately for horizontal and vertical dimensions. The conditions of 20 pixels *d*_*max*_ in the horizontal direction and 20 pixels *d*_*max*_ in the vertical direction were duplicated.

**TABLE 1:** *d*_*max*_ (maximum deformation magnitudes) for horizontal and vertical dimensions.

	*d*_*max*_ (pixels)													
Horizontal	20	20	20	20	20	20	20	5	10	15	20	25	30	35
Vertical	5	10	15	20	25	30	35	20	20	20	20	20	20	20
SS ratio in HOVI	4	2	1.333	1	0.8	0.667	0.571	0.25	0.5	0.75	1	1.25	1.5	1.75
SS ratio in HIVO	0.25	0.5	0.75	1	1.25	1.5	1.75	4	2	1.333	1	0.8	0.667	0.571

*A SS ratio means a stretching-to-squashing ratio of deformation magnitudes.*

#### Manipulation of Deformation Vectors

We manipulated the direction of deformation vectors between horizontal and vertical dimensions. In one condition, that is, the horizontally-outward-vertically-inward (HOVI) condition, the deformation vectors were outward and inward for horizontal and vertical dimensions, respectively. In the other condition, that is, the horizontally-inward-vertically-outward (HIVO) condition, the deformation vectors were inward and outward for horizontal and vertical dimensions, respectively. Apart from the direction of deformation vectors, we also examined the effect of symmetricity of motion vectors between horizontal and vertical deformations. In the Symmetry condition, both the vectors of horizontal (vertical) and vertical (horizontal) deformations were inward and outward, respectively. In the Asymmetry condition, however, the vectors of horizontal and vertical deformations were asymmetric, as shown in Figure 3B. In the Asymmetry condition of the HOVI condition, the vertical deformation had a downward vector only. In the Asymmetry condition of the HIVO condition, the horizontal deformation had a rightward vector only.

### Procedure

The experiment was programmed by using jsPsych (de Leeuw, 2015). Observers participated in the experiment in their own environments with personal computers. Neither observation distances nor screen sizes were controlled. Although the presentation accuracy was not also measured, it has been reported that stimulus timing control with jsPsych was sufficient to conduct online psychological experiments (Bridges et al., 2020). The only instruction about the environment was that they should not perform the task in a dark environment in order to reduce potential epilepsy. After viewing the sequence as shown in Figure 2, the participants judged the apparent force direction pattern; here the force direction means the perceived direction in which the material is stretched or squashed. Specifically, in the HOVI condition, they were asked to judge whether the material was horizontally stretched or vertically squashed. In the HIVO condition, they were asked to judge whether the material was horizontally squashed or vertically stretched. They reported their judgment by pressing the assigned keys. Participants were divided into four groups, each performing either of the four conditions (HOVI-Symmetry, HOVI-Asymmetry, HIVO-Symmetry, and HIVO-Asymmetry). Each participant performed 140 trials consisting of 14 *d*_*max*_ conditions (as shown in Table 1) and 10 repetitions.

## Results

**FIGURE 4:**
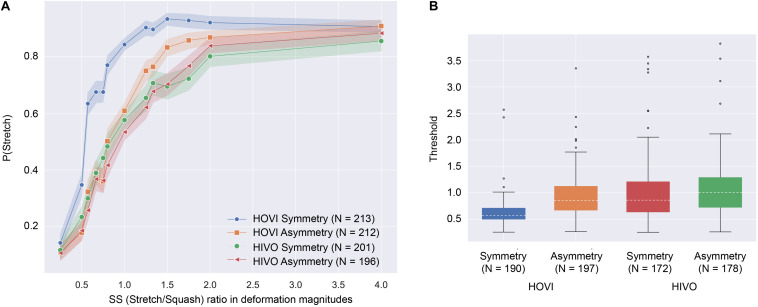
**(A)** Proportion of trials reporting stretching as a function of SS ratio in deformation magnitudes. The shaded areas show 95% confidence intervals among the participants. **(B)** The box plot of the threshold to report stretching in each condition. The dotted lines in the boxes show the median threshold.

We calculated the stretching-to-squash (SS) ratio of deformation magnitudes, as shown in Table 1. Figure 4A plots the proportion of trials in which the participants reported stretching as a function of SS ratio in deformation magnitudes, which was calculated by the following formula,

(3) $$\text{SS}\,\text{ratio}=\frac{\text{Stretch}\,\text{deformation}\,\text{magnitude}}{\text{Squash}\,\text{deformation}\,\text{magnutude}}$$

The SS ratio differs from the Poisson’s ratio in several ways. First, if the original material shape before deformation is not square, the SS ratio takes on a different value than the Poisson’s ratio. Second, if the material is assumed to be squashing, the SS ratio is equal to the Poisson’s ratio, but if the material is assumed to be stretching, the Poisson’s ratio is the inverse of the SS ratio in our stimulus setting. Third, as stated in the “Introduction,” the Poisson’s ratio of natural materials does not exceed 0.5. Because the SS ratio is not subject to the laws of physics, it exceeds 0.5. We chose to use the SS ratio instead of the Poisson’s ratio in this study because we would like to examine how the relative magnitude of image deformation between the horizontal and vertical dimensions contributed to the judgment of the stretching-squashing events in the Poisson effect. When the SS ratio was 1, the judgment of stretching/squashing events would be ambiguous if the observers relied their judgments on the relative magnitude of image deformation. We also examined how the judgment was biased by the vector symmetricity and vector direction. As shown in Figure 4A, the proportion of reporting stretching increased with the SS ratio. The pattern of results suggests that the participants used the SS ratio to determine the pattern of force direction. Importantly, the function of the proportion in the HOVI Symmetry condition shifted leftward from the functions in the other three conditions. To ascertain whether the shift of the function was statistically significant, we decided to fit a psychometric function (i.e., a cumulative normal function) to the proportion and calculated the threshold for the report of stretching. We used psignifit for python (Schütt et al., 2016) for the calculation of the threshold. Some subjects’ thresholds were outside the stimulus range (0.25–4 of the SS ratio). Because these outlier data likely came from the unreliable fitting of the psychometric function to the data, they were excluded from the further analysis. The number of data excluded from the analysis was 23, 15, 29, and 18 for the HOVI Symmetry, the HOVI Asymmetry, the HIVO Symmetry, and the HIVO Asymmetry conditions, respectively. Figure 4B shows the box plot of the threshold for each condition. Using the threshold, we conducted a two-way ANOVA with the vector direction (HOVI vs. HIVO) and the vector symmetricity (Symmetry vs. Asymmetry) as between-subject factors. The main effect of the vector direction was significant [*F*(1,734) = 64.582, *p* < 0.0001, η*_*p*_^2^* = 0.08]. The main effect of the vector symmetricity was not significant [*F*(1,734) = 30.675, *p* = 0.0537, η*_*p*_^2^* = 0.04]. Interaction between the two factors was significant [*F*(1,734) = 15.020, *p* = 0.0001, η*_*p*_^2^* = 0.02]. The simple main effect of the vector direction showed that the threshold in the HOVI condition was significantly lower than the one in the HIVO condition for the Symmetry condition [*F*(1,818) = 47.956, *p* < 0.0001, η*_*p*_^2^* = 0.0554] and the Asymmetry condition [*F*(1,818) = 4.6203, *p* = 0.0319, η*_*p*_^2^* = 0.0056]. The simple main effect of the vector symmetricity showed that the threshold in the Symmetry condition was significantly lower than the one in the Asymmetry condition for the HOVI condition [*F*(1,818) = 14.456, *p* = 0.0002, η*_*p*_^2^* = 0.0174]. On the other hand, there was no significant difference in the threshold between the Symmetry and Asymmetry conditions in the HIVO condition [*F*(1,818) = 0.9749, *p* = 0.323, η*_*p*_^2^* = 0.0012].

Additionally, we conducted a one-sample *t*-test to check whether the threshold deviated from 1. When the SS ratio was 1, the magnitude of deformation was equivalent between squashing and stretching. If the visual system purely used the relative magnitude of image deformation between the horizontal and vertical dimensions, the threshold should not deviate from 1. In the HOVI Symmetry condition, the threshold was significantly lower than 1 [*t*(189) = −20.342, *p* < 0.001]. In the HOVI Asymmetry condition, the threshold was significantly lower than 1 [*t*(196) = −2.370, *p* = 0.018]. In the HIVO Symmetry condition, the threshold was not significantly deviated from 1 [*t*(171) = 0.315, *p* = 0.753]. In the HIVO Asymmetry condition, the threshold was not significantly deviated from 1 [*t*(178) = 1.800, *p* = 0.073].

## Discussion

We examined how the human observers judged the force direction in the Poisson effect.

Consistent with our prediction, the proportion of reports for stretching relied strongly on the SS ratio (Figure 4A). The results indicate that the observers used the relative magnitude of deformation between the horizontal and vertical dimensions to determine the force direction in the Poisson effect. When the SS ratio is greater than 1, the Poisson ratio is also greater than 1 if squashing events are assumed. On the other hand, when stretching events are assumed, the Poisson ratio becomes smaller than 1, unlike the SS ratio. When the SS ratio is less than 1, squashing events tend to be reported. On the other hand, when the SS ratio exceeded 1, the proportion of reporting stretching events increased. This result can be interpreted to mean that observers reported events in which the Poisson ratio was smaller. Since the value of Poisson’s ratio exceeds 0.5 in some conditions, it is unlikely that the observer interpreted the stimulus according to physical laws. On the other hand, it implies that the observer is somehow highly sensitive to this relative amount of deformation, which needs to be investigated further.

On the other hand, we observed an interesting anisotropy in the force direction judgment. In both the HOVI Symmetry and HOVI Asymmetry conditions, the threshold significantly deviated from 1. The results indicate that the judgment was biased toward stretching when horizontally outward deformation was given to the noise square. It is unclear why this sort of anisotropy occurred in the judgment of the Poisson effect. In the HOVI condition stimuli, the vertical deformation vector was inward while the horizontal deformation vector was outward. In general, inward and outward motion vectors are involved with contraction and expansion motion. Previous studies investigating complex motion perception have been shown that the detection and interpretation of expansion motion were prioritized over those of contraction motion (Perrone, 1986; Takeuchi, 1997; Lewis and McBeath, 2016). If the expansion-contraction anisotropy were the source of the anisotropy in the force-direction judgment of the Poisson effect, we should have observed a similar kind of the judgment bias in the HIVO condition because in the HIVO condition stimuli, the vertical and horizontal deformation vectors were outward and inward, respectively, and hence, both the expansion and contraction motion were contained. We suggest that the factor other than the expansion-contraction anisotropy contributed to the anisotropy in the force-direction judgment for the Poisson effect.

The vector asymmetry also contributed to the determination of the judgment bias. The threshold in the HOVI Symmetry condition was significantly lower than the one in the HOVI Asymmetry condition. The results are consistent with our hypothesis that the asymmetric vector pattern, which was involved with the situation, as shown in Figure 1D, would bias the participant’s responses toward squashing. On the other hand, the threshold in the HIVO condition was not significantly different between the Symmetry and Asymmetry conditions. Thus, the vector symmetricity seems to be effective only in the HOVI condition that is involved with horizontal stretching and vertical squashing.

In this on-line experiment, neither the observation distance nor the screen size was controlled. Therefore, it is an open question whether the stimulus size can affect the stretching/squashing of the judgment in the Poisson effect. Here, we would like to discuss some perspectives to support that manipulation of viewing distance or stimulus magnitude is not important in subjectively determining squash-stretch events. First, the variation of viewing distance and/or stimulus sizes equally influences the deformation magnitude of the horizontal and vertical dimensions in a retinal image, and this will not affect the ratio between them. Second, in the real world, the Poisson effect can occur for the objects of various sizes. When the brain learns the statistical properties of the Poisson effect, learning will not depend on the size of objects. This is because the Poisson effect occurs at various scales, so the learning is possibly independent of object sizes. Consistent with this idea, our results showed that the reported proportion of stretching/squashing judgment was stable across the observers (as shown by the 95% confidence intervals in Figure 4A), even though the observers likely performed the task at different observation distances and different screen sizes. The results indicate that the judgment of the stretching/squashing is not so susceptible to stimulus sizes in a retinal image. On the other hand, the results do not mean that the stimulus size has no influence on the perceptual judgment of the Poisson effect. It is important to investigate in detail the effect of stimulus parameters as like stimulus size under controlled experimental settings.

In this study, two-dimensional texture rectangles were used as stimuli. As described in the Introduction, the Poisson effect occurs along the three spatial axes (i.e., one longitudinal and two orthogonal axes). Although the physical behavior of the Poisson effect is often described by the two-dimensional pattern, the Poisson effect of a three-dimensional material causes a change in visual signals useful to a human observer who judges the material properties. For example, deformation of the three-dimensional surface often results in a local change in the three-dimensional waveform, which results in a change in the specular and/or refractive pattern. Such changes in optical information are powerful clues to material perception (Kawabe and Nishida, 2016; Paulun et al., 2017; Schmidt et al., 2017; Schmid and Doerschner, 2018). The texture of the material surface is also a clue to the three-dimensional shape. It is a future problem to examine the effect of these surface information on the perception judgment of the Poisson effect.

As speculation, we suggest that there may be an internalized assumption or “a prior” about the relationship between the force direction and resultant image deformation. For example, the brain may have an assumption that stretching is related to horizontal vectors. For example, the stretching behavior, such as stretching the cloth, is mainly done with both hands on the horizontal axis. The assumption may underlie the significant difference of threshold from 1 in the HOVI condition. The vector asymmetry was also effective in the HOVI condition only. This may also come from a different assumption that the vector asymmetry, as shown in Figure 1D, likely occurs in the situation wherein an object is squashed from above. It is worth investigating whether such assumptions work in judging the force direction in future studies. By analyzing image (and/or motion) statistics in natural scene movies, it is possible to check whether stretching events actually involves horizontal deformation vectors. Psychophysical testing in combination with Bayesian modeling may be able to check whether the knowledge of the deformation statistics influences the judgment of stretching/squashing events in the Poisson effect.

## Data Availability Statement

The raw data supporting the conclusions of this article will be made available by the authors, without undue reservation.

## Ethics Statement

The studies involving human participants were reviewed and approved by NTT Communication Science Laboratories Research Ethics Committee. The patients/participants provided their written informed consent to participate in this study.

## Author Contributions

TK designed and conceived this study, wrote the manuscript, and finalized the manuscript for submission.

## Conflict of Interest

The author declares that the research was conducted in the absence of any commercial or financial relationships that could be construed as a potential conflict of interest.
